# Supplementary material for: Glutamine metabolism inhibition has dual immunomodulatory and antibacterial activities against Mycobacterium tuberculosis
Source: Nat Commun. 2023 Nov 16;14:7427. doi: 10.1038/s41467-023-43304-0 (PMC10654700; doi:10.1038/s41467-023-43304-0)
Supplement: Supplementary file 1 — Supplementary Information [file 41467_2023_43304_MOESM1_ESM.pdf]

SUPPLEMENTARY INFORMATION

Glutamine metabolism inhibition has dual immunomodulatory and antibacterial activities against *Mycobacterium tuberculosis*

Sadiya Parveen[1], Jessica Shen[1], Shichun Lun[1], Liang Zhao[2], Jesse Alt[3], Benjamin Koleske[1], Robert D. Leone[2,4], Rana Rais[5,6,7], Jonathan D. Powell[4, §], John R. Murphy[1], Barbara S. Slusher[2,3,4,5,6,7,8,9], William R. Bishai[1]*

[1]Center for Tuberculosis Research, Department of Medicine, Johns Hopkins School of Medicine, Baltimore, MD, USA

[2]Department of Oncology, Johns Hopkins School of Medicine, Baltimore, MD, USA

[3]Johns Hopkins University, Baltimore, MD, USA.

[4]The Bloomberg-Kimmel Institute for Cancer Immunotherapy, Johns Hopkins School of Medicine, Baltimore, MD, USA

[5]Johns Hopkins Drug Discovery, Johns Hopkins School of Medicine, Baltimore, MD, USA.

[6]Department of Neurology, Johns Hopkins School of Medicine, Baltimore, MD, USA.

[7]Department of Pharmacology and Molecular Sciences, Johns Hopkins School of Medicine, Baltimore, MD, USA.

[8]Department of Neuroscience, Johns Hopkins School of Medicine, Baltimore, MD, USA.

[9]Department of Psychiatry and Behavioral Sciences, Johns Hopkins School of Medicine, Baltimore, MD, USA.

§ Current address: Calico, S. San Francisco, CA

Keywords: Tuberculosis, TB drug, Immunotherapy, Glutamine inhibition, Host-directed therapy

*Corresponding author

William R. Bishai

Johns Hopkins School of Medicine

1550 Orleans St, CRB2 Rm 108

Baltimore, MD 21287

Phone: 4109553507

wbishai1@jhmi.edu

SUPPLEMENTARY FIGURES AND FIGURE LEGENDS

Fig S1.

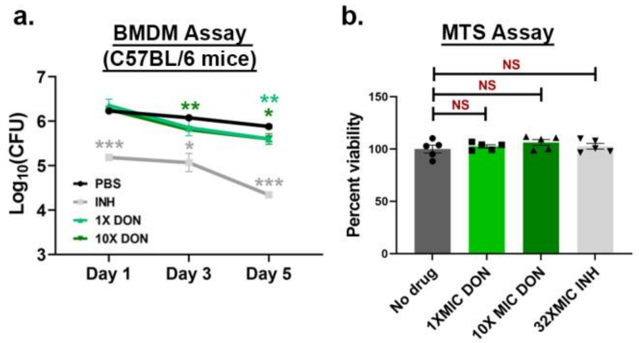

Fig S1. The effect of JHU083 on the intracellular survival of Mtb and on bone-marrow derived macrophage (BMDM) viability. BMDMs were harvested from the femurs of 8-12 weeks old C57BL6 mice (n=10/experiment), activated using IFNγ. **(a)** IFNγ-activated BMDMs were infected with *Mtb* H37Rv at an MOI of 2 (n=3 wells/group). They were then treated with 1X and 10X MIC concentrations of DON assuming a MIC value of 1 mg/ml. Isoniazid (INH) was used as the positive control. The cells were lysed at indicated time points and plated on 7H11 selection plates. **(b)** BMDM viability as assessed by an MTS assay was performed after 5 days of daily drug treatment at the indicated concentration (n=5 wells/group). The experiment was performed in triplicate. Statistical significance was calculated using a two-tailed student t-test considering unequal distribution. The exact p-values are provided in the Source Data file. *<0.05, **<0.01, ***<0.001. CFU stands for colony-forming units. All the experiments were performed in triplicates. MTS stands for 3-(4,5-Dimethylthiazol-2-yl)-5-(3-carboxymethoxyphenyl)-2-(4-sulfophenyl)-2H-tetrazolium salt. CFU stands for colony-forming units. NS stands for non-significant change, p-value was >0.05.

Fig S2.

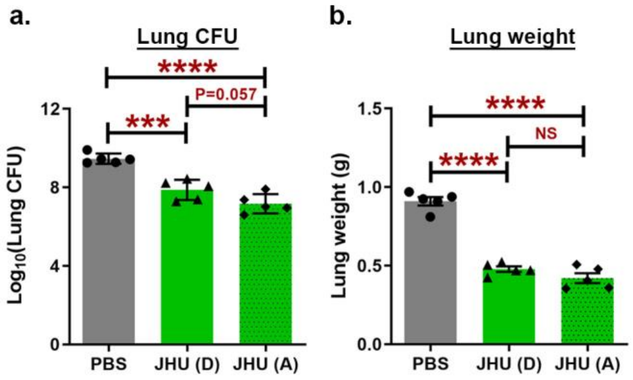

Fig S2. Both daily and alternate JHU083 dosing regimen are effective in vivo. 129S2 mice (n=5/group) were aerosol infected with ~200-300 CFU of *Mtb* H37Rv. Two different dosing regimens; (1) Daily (JHU-D; 1 mg/kg dose per day for the first week, followed by 0.3 mg/kg daily 5/7 dosing M-F) and (2) Alternate (JHU-A; 1 mg/kg dose per day for the first week, followed by 3/7 dosing with 1 mg/kg on Mon, Wed and Fri) were administered. The mice were sacrificed at day 0 and week 5 post-infection/treatment. The lungs were harvested, homogenized, serially diluted, and plated on 7H11 selection plates. After 21-25 days, colonies were counted, and counts were transformed into log_{10} values and plotted. Graphs depicting the effect of both dosing regimen upon **(a)** lung bacillary burden and **(b)** lung weight at 5 weeks post infection/treatment. Data is plotted as mean ± SEM. Statistical significance was calculated using a two-tailed student t-test considering unequal distribution. The exact p-values are provided in the Source Data file. *<0.05. CFU stands for colony-forming units. CFU stands for colony-forming units. NS stands for non-significant change, p-value was >0.05. The experiment was performed twice.

Fig S3.

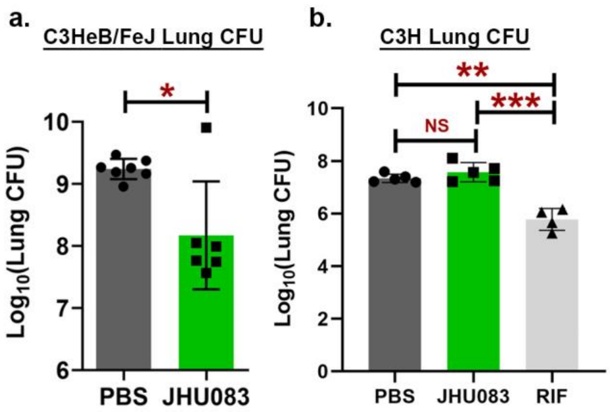

Fig S3. Impact of JHU083 treatment on lung bacillary burden in *Mtb*-infected C3HeB/FeJ and C3H mice. C3HeB/FeJ (n=7/group) and C3H mice (n=5/group) were aerosol-infected with 150-200 CFU of *Mtb* H37Rv. The mice were then treated with JHU083 via oral gavage starting one day after infection. 1 mg/kg JHU083 was given daily for the first 5 days and then the dose was reduced to 0.3 mg/kg daily (5/7, M-F). The mice were sacrificed on day 0 and week 5 post-infection/treatment. The lungs were harvested, homogenized, serially diluted, and plated on 7H11 selection plates. After 21-25 days, colonies were counted, and counts were transformed into $\log_{10}$ values and plotted. Lung bacillary burden in **(a)** C3HeB/FeJ mice at 4.5 weeks post infection/treatment and **(b)** C3H mice at 5 weeks post infection/treatment. Data is plotted as mean ± SEM. Statistical significance was calculated using a two-tailed student t-test considering unequal distribution. The exact p-values are provided in the Source Data file. *<0.05. CFU stands for colony-forming units. CFU stands for colony-forming units. NS stands for non-significant change, , p-value was >0.05. One mouse each from JHU083-treated C3HeB/Fej and RIF-treated C3H groups died prematurely. The experiment was performed twice.

Fig S4.

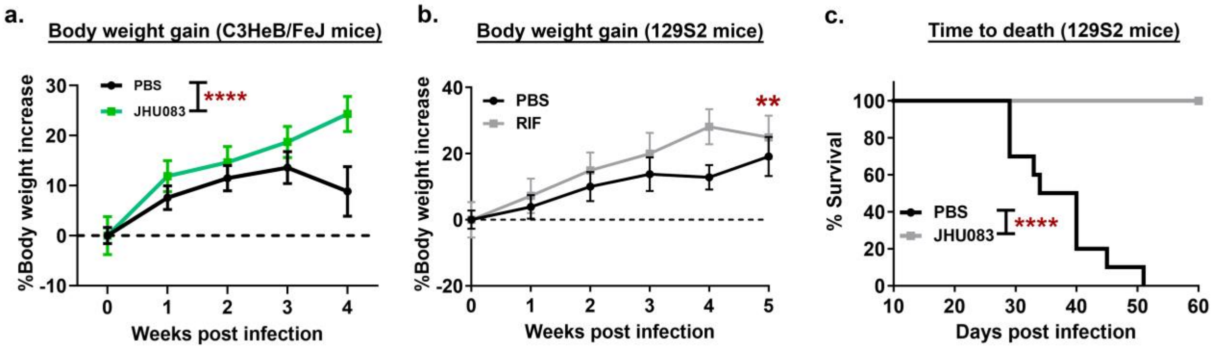

Fig S4. JHU083 and RIF administration improved body weight and survival of *Mtb*-infected mice. 129S2 or C3HeB/FeJ mice were aerosol-infected with 200-300 CFU of *Mtb H37Rv*. Next day of the infection, 1 mg/kg of JHU083 was given daily for the first five days, and then the dose was reduced to 1 mg/kg on alternate days (3/7, M-F). 12.5 mg/kg RIF was given daily orally for 5-weeks. The mice were then treated with JHU083 12.5 mg/kg RIF daily via oral gavage starting one day after infection. The mice were sacrificed at day 0 and week 5 post-infection/treatment. Graphs depict (a) body weight gain in C3HeB/Fej mice (n=10/group) treated with PBS or JHU083 and, (b) body weight gain in 129S2 mice (n=5/group) treated with PBS and RIF. (c) Survival of 129S2 mice (n=10/group) treated with RIF or PBS. Data is plotted as mean ± SEM. For body weight, a two-way Anova test was used for calculating statistical significance. For survival curve, statistical significance was calculated using both Log-rank (Mantal-Cox) and Gehan-Breslow-Wilcoxon tests. The exact p-values are provided in the Source Data file. **<0.01, ****<0.0001. The experiment was performed twice.

Fig S5

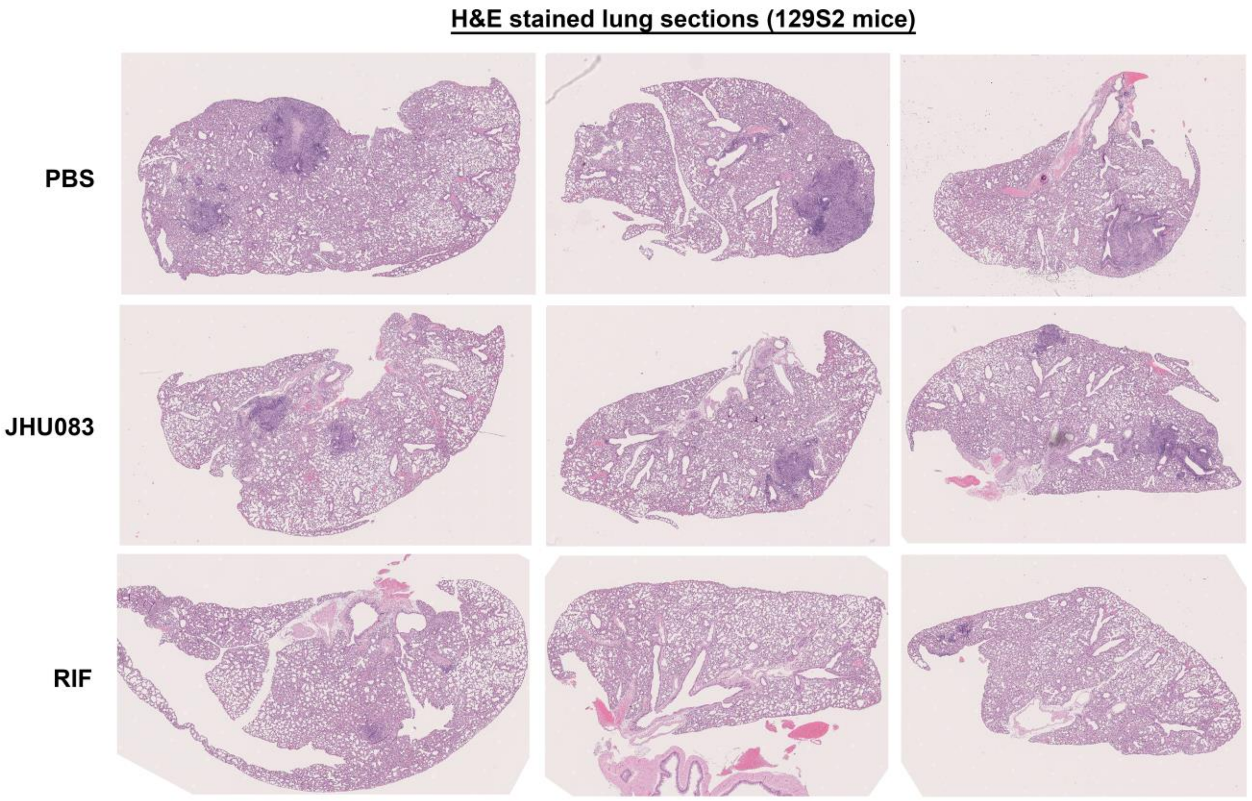

Fig S5. The histopathology of the lungs isolated from 129S2 mice infected with *Mtb* H37Rv at week 5 post-infection/treatment. 129S2 or C3HeB/FeJ mice (n=3/group) were aerosol infected with ~200-300 CFU of *Mtb H37Rv*. The mice were treated with JHU083 or RIF via oral route one day after infection. 1 mg/kg JHU083 was given daily for the first five days, and then the dose was reduced to 0.3 mg/kg daily (5/7, M-F). The lungs were formalin fixed, sectioned, and stained with hematoxylin and eosin. All the experiments were repeated at least twice.

Fig S6.

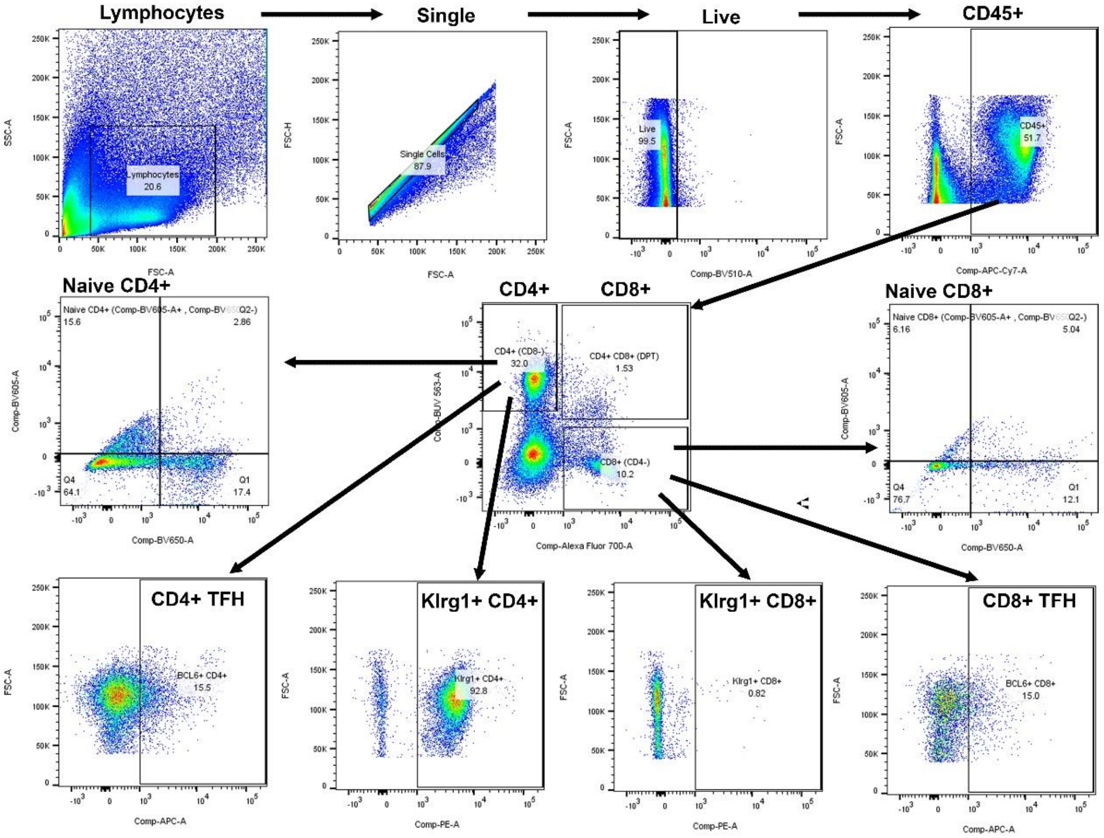

Fig S6. Two-dimensional gating strategies for flow cytometrical identification of T-cell subsets. We excluded doublets and debris and gated on single live $CD45^{+}$ cells. We identified $CD4^{+}$ T-cells ($CD45^{+}$ $CD8^{-}$ $CD4^{+}$), $CD8^{+}$ T-cells (CD45+ $CD4^{-}$ $CD8^{+}$), Naïve $CD4^{+}$ T-cells ($CD45^{+}$ $CD8^{-}$ $CD4^{+}$ $CD44^{-}$ $CD26L^{+}$), Naïve $CD8^{+}$ T-cells ($CD45^{+}$ $CD8^{+}$ $CD4^{-}$ $CD44^{-}$ $CD26L^{+}$), Follicular helper $CD4^{+}$ T-cells ($CD45^{+}$ $CD8^{-}$ $CD4^{+}$ $BCL6^{+}$), Follicular helper $CD8^{+}$ T-cells ($CD45^{+}$ $CD8^{+}$ $CD4^{-}$ $BCL6^{+}$), $Klrg1^{+}$ $CD4^{+}$ T-cells ($CD45^{+}$ $CD8^{-}$ $CD4^{+}$ $Klrg1^{+}$), $Klrg1^{+}$ $CD8^{+}$ T-cells ($CD45^{+}$ $CD8^{+}$ $CD4^{-}$ $Klrg1^{+}$). All flow antibodies were titrated to identify the concentration with maximum specificity coupled with minimum possible spillover. The gating strategy was defined using single-stain and FMO controls (for low-expression markers).

Fig S7.

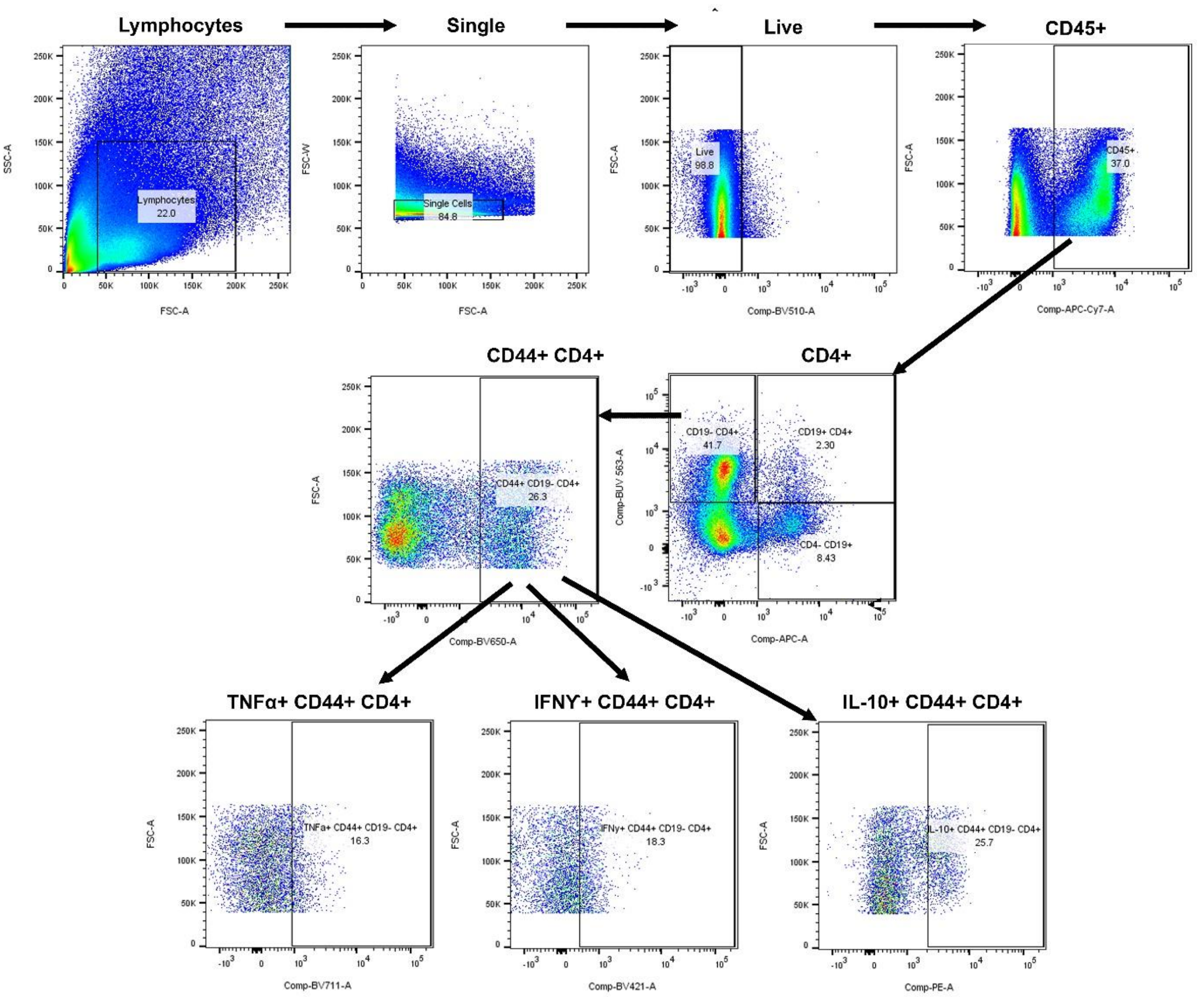

Fig S7. Two-dimensional gating strategies for flow cytometrical identification of cytokine-producing T-cell subsets. We excluded doublets and debris and gated on single live CD45+ cells. We identified $CD4^+$ T-cells ($CD45^+$ $CD19^-$ $CD4^+$), activated $CD4^+$ T-cells ($CD45^+$ $CD19^-$ $CD44^+$ $CD4^+$), $TNF\alpha^+$ activated $CD4^+$ T-cells ($CD45^+$ $CD19^-$ $CD44^+$ $CD4^+$ $TNF\alpha^+$), $IFN\gamma^+$ activated $CD4^+$ T-cells ($CD45^+$ $CD19^-$ $CD44^+$ $CD4^+$ $IFN\gamma^+$) and IL-10^+ activated $CD4^+$ T-cells ($CD45^+$ $CD19^-$ $CD44^+$ $CD4^+$ $IL10^+$). All flow antibodies were titrated to identify the concentration with maximum specificity coupled with minimum possible spillover. The gating strategy was defined using single-stain and FMO controls (for low-expression markers).

Fig S8.

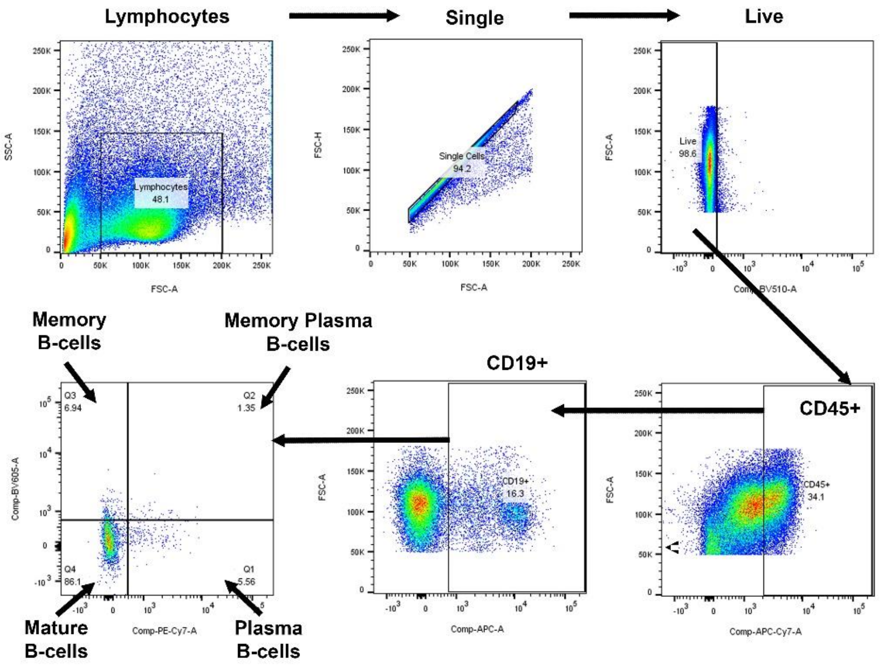

Fig S8. Two-dimensional gating strategies for flow cytometrical identification of B cell subsets. We excluded doublets and debris and gated on single live $CD45^+$ cells. We identified total T-cells ($CD45^+$ $CD19^+$), mature B-cells ($CD45^+$ $CD19^+$ $CD27^-$ $CD138^-$), memory B-cells ($CD45^+$ $CD19^+$ $CD27^+$ $CD138^-$), plasma cells ($CD45^+$ $CD19^+$ $CD27^-$ $CD138^+$), plasma memory B-cells ($CD45^+$ $CD19^+$ $CD27^+$ $CD138^+$). All flow antibodies were titrated to identify the concentration with maximum specificity coupled with minimum possible spillover. The gating strategy was defined using single-stain and FMO controls (for low-expression markers).

Fig S9.

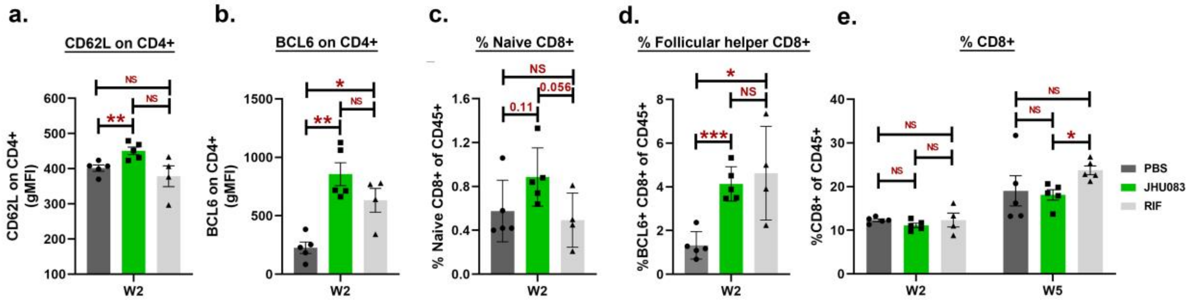

Fig S9. Effect of JHU083 administration upon T-cells in the lungs at weeks 2 and 5. As described in Fig 2a, *Mtb*-infected 129S2 mice were treated with JHU083 and RIF every day starting day 1 post-infection. The mice were sacrificed at week 2 and week 5, and the lungs were harvested. Single cell suspensions of the lungs from all three groups were stained with appropriate antibodies and analyzed using multicolor-flow cytometry (n=5/group except RIF-treated group in graph panels a-d where n=4). We found <u>differences</u> in the **(a)** CD62L expression upon $CD4^+$ T-cells, **(b)** BCL6 expression upon $CD4^+$ T-cells, **(c)** frequency of naïve $CD8^+$ T-cells, **(d)** frequency of follicular helper $CD8^+$ T-cells and, **(e)** total $CD8^+$ T-cells The X-axis shows the timepoint at which the lungs were harvested for the flow cytometry analysis. Data were plotted as Mean ± SEM and are shown as the frequency of $CD45^+$ population. gMFI stands for geometric mean fluorescence intensity and was used to define the expression of the individual markers upon the indicated cell types. Statistical significance was calculated using a two-tailed student t-test considering unequal distribution. The exact p-values are provided in the Source Data file. *<0.05, **<0.01, ***<0.001. CFU stands for colony-forming units. NS stands for non-significant change, p-value was >0.05. The experiment was repeated two times.

Fig S10.

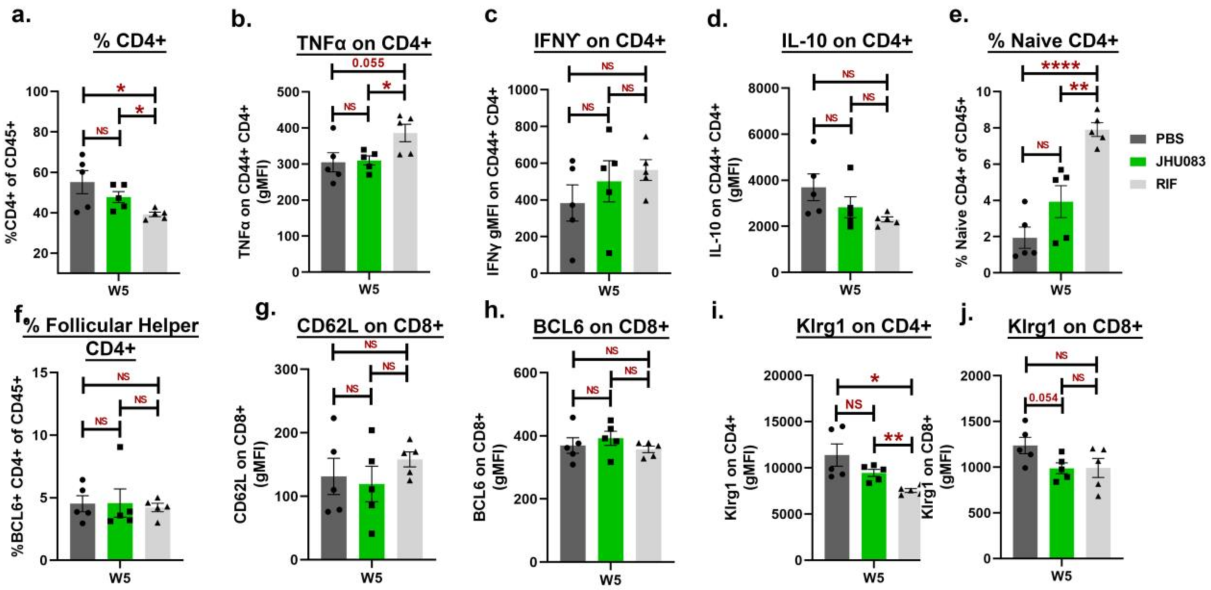

Fig S10. Effect of JHU083 administration upon T-cells in the lungs at week 5. As described in Fig 2a, *Mtb*-infected 129S2 mice (n=5/group) were treated with JHU083 and RIF every day starting day 1 post-infection. The mice were sacrificed at week 2 and week 5, and the lungs were harvested. Single cell suspensions of the lungs from all three groups were stained with appropriate antibodies and analyzed using multicolor-flow cytometry (n=4-5). We found no difference in the **(a)** $CD4^+$ T-cell frequency, **(b)** TNFα expression upon activated $CD4^+$ T-cells, **(c)** IFNγ expression upon activated $CD4^+$ T-cells, **(d)** IL-10 expression upon activated $CD4^+$ T-cells, **(e)** Naïve $CD4^+$ T-cells, **(f)** Follicular helper T-cells, **(g)** CD62L expression on $CD8^+$ T-cells, **(h)** BCL6 expression on $CD8^+$ T-cells, **(i)** Klrg1 expression on $CD4^+$ T-cells, **(j)** Klrg1 expression on $CD8^+$ T-cells. The X-axis shows the timepoint at which the lungs were harvested for flow cytometry analysis. Data were plotted as Mean ± SEM and are shown as the frequency of $CD45^+$ population. gMFI stands for geometric mean fluorescence intensity and was used to define the expression of the individual markers upon the indicated cell types. gMFI was mostly used for low abundance cell surface markers and transcription factors. Statistical significance was calculated using a two-tailed student t-test considering unequal distribution. The exact p-values are provided in the Source Data file. *<0.05, **<0.01, ***<0.001, ****<0.0001. CFU stands for colony-forming units. NS stands for non-significant change, p-value was >0.05. The experiment was repeated twice.

Fig S11.

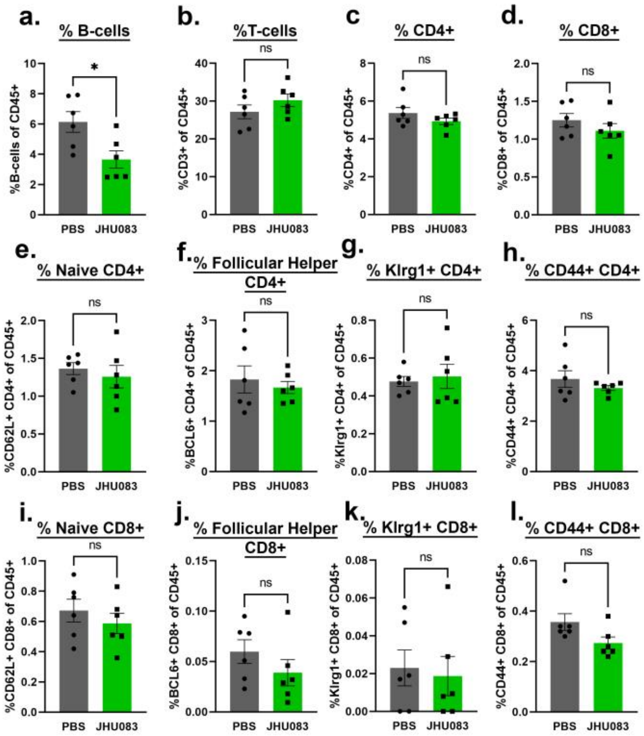

Fig S11. Effect of JHU083 administration upon T-cell subsets in uninfected 129S2 mice lungs at week 2. Uninfected six to ten weeks old female 129S2 mice (n=6/group) were treated with JHU083 every day. The mice were sacrificed at week 2, and the lungs were harvested. Single cell suspensions of the lungs from all three groups were stained with appropriate antibodies and analyzed using multicolor-flow cytometry. We found **(a)** reduced frequency of B-cells. While there was no difference in the frequency of **(b)** $CD3^+$ T-cells, **(c)** $CD4^+$ T-cells, **(d)** $CD8^+$ T-cells, **(e) Naïve** $CD4^+$ T-cells, **(f)** Follicular helper $CD4^+$ T-cells, **(g)** proliferating $Ki67^+$ $CD4^+$ T-cells, **(h)** activated $CD44^+$ $CD4^+$ T-cells, **(i)** Naïve $CD8^+$ T-cells, **(j)** Follicular helper $CD8^+$ T-cells, **(k)** proliferating $Ki67^+$ $CD8^+$ T-cells and, **(l)** activated $CD44^+$ $CD8^+$ T-cells. Data were plotted as Mean ± SEM and are shown as the frequency of $CD45^+$ population. Statistical significance was calculated using a two-tailed student t-test considering unequal distribution. The exact p-values are provided in the Source Data file. *<0.05. CFU stands for colony-forming units. NS stands for non-significant change, p-value was >0.05. The experiment was performed twice.

Fig S12.

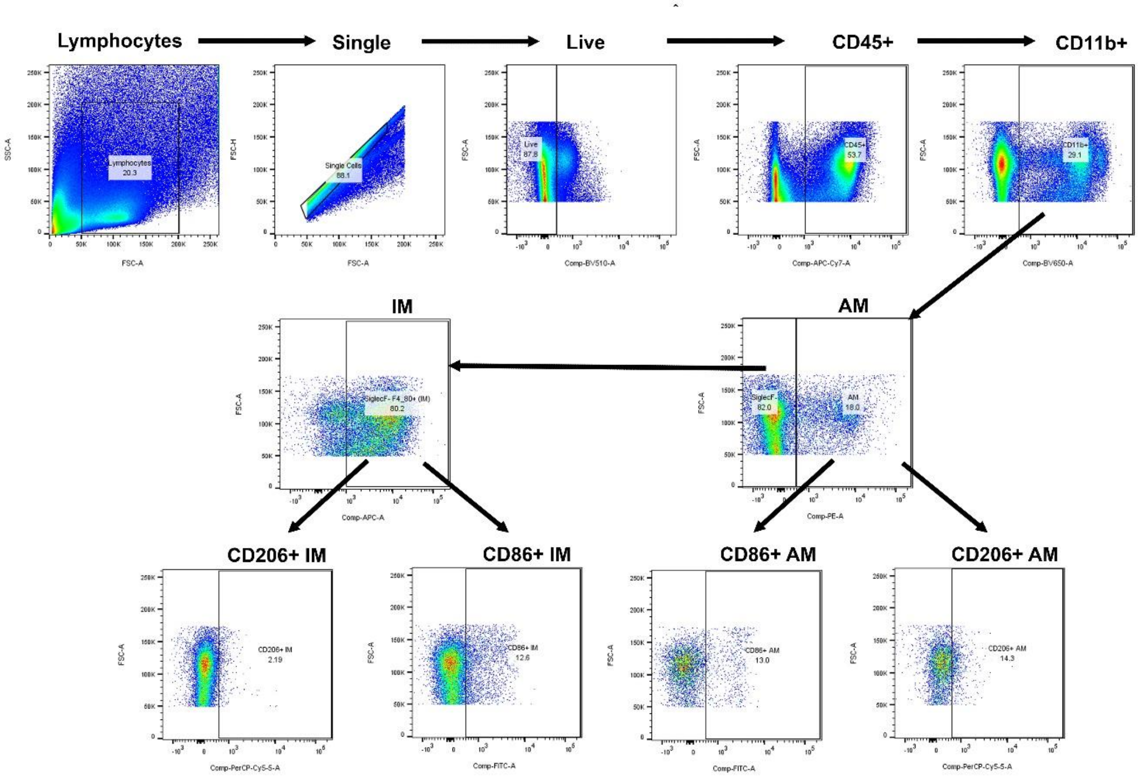

Fig S12. Two-dimensional gating strategies for flow cytometrical identification of myeloid cell subsets. We excluded doublets and debris and gated on single live CD45+ cells. We identified myeloid cells (CD45$^+$ CD11b$^+$), alveolar macrophages (AM; CD45$^+$ CD11b$^+$ SiglecF$^+$), interstitial macrophages (IM; CD45$^+$ CD11b$^+$ SiglecF$^-$ F4/80$^+$), CD86$^+$ alveolar macrophages (AM; CD45$^+$ CD11b$^+$ SiglecF$^+$ CD86$^+$), CD206$^+$ alveolar macrophages (AM; CD45$^+$ CD11b$^+$ SiglecF$^+$ CD206$^+$), CD86$^+$ interstitial macrophages (IM; CD45$^+$ CD11b$^+$ SiglecF$^-$ F4/80$^+$ CD86$^+$), CD206$^+$ interstitial macrophages (IM; CD45$^+$ CD11b$^+$ SiglecF$^-$ F4/80$^+$ CD206$^+$). All flow antibodies were titrated to identify the concentration with maximum specificity coupled with minimum possible spillover. The gating strategy was defined using single-stain and FMO controls (for low-expression markers).

Fig S13.

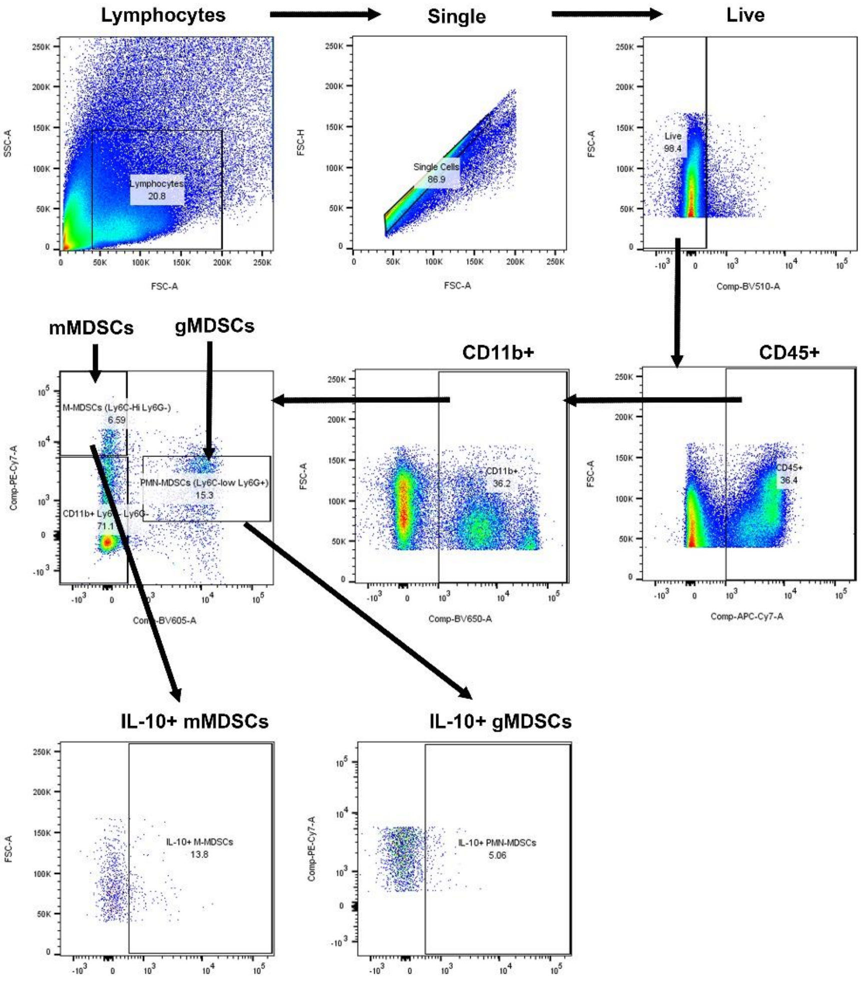

Fig S13. Two-dimensional gating strategies for flow cytometrical identification of MDSC subsets. We excluded doublets and debris and gated on single live $CD45^{+}$ cells. We identified myeloid cells (CD45+ $CD11b^{+}$), monocytic MDSCs ($CD45^{+}$ $CD11b^{+}$ $Ly6G^{-}$ $Ly6C^{High}$), IL-10^{+} monocytic MDSCs ($CD45^{+}$ $CD11b^{+}$ $Ly6G^{-}$ $Ly6C^{High}$, IL-10^{+}), granulocytic MDSCs ($CD45^{+}$ $CD11b^{+}$ $Ly6G^{+}$ $Ly6C^{low}$), granulocytic MDSCs ($CD45^{+}$ $CD11b^{+}$ $Ly6G^{+}$ $Ly6C^{low}$, IL-10^{+}). All flow antibodies were titrated to identify the concentration with maximum specificity coupled with minimum possible spillover. The gating strategy was defined using single-stain and FMO controls (for low-expression markers).

Fig S14.

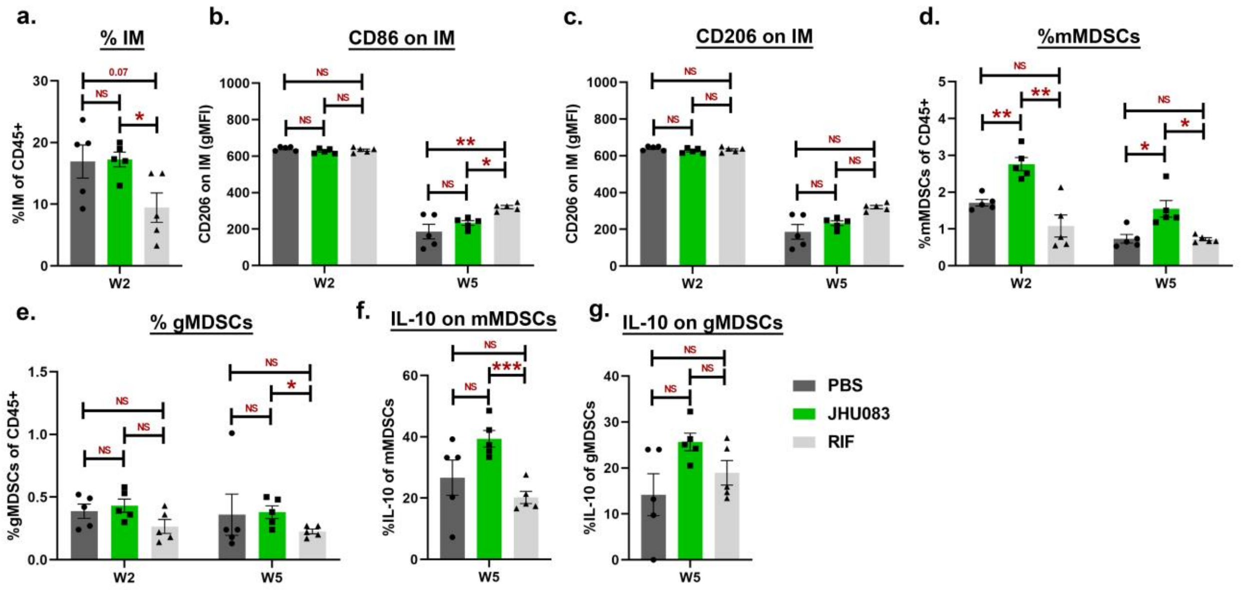

Fig S14. Effect of JHU083 treatment upon myeloid cell subsets. As described in Fig 2a, *Mtb*-infected female 129S2 mice (n=5/group) were treated with JHU083 and RIF every day starting day 1 post-infection. The mice were sacrificed at week 2 and week 5, and the lungs were harvested. Single cell suspension of the lungs from all three groups were stained with appropriate antibodies and analyzed using multicolor-flow cytometry (n=5). Details are provided in "Methods" section. We found no difference in the **(a)** interstitial macrophages (IM), **(b)** CD86 expression on IM, **(c)** CD206 expression on IM, **(d)** monocytic myeloid-derived suppressor cells (mMDSCs), **(e)** granulocytic myeloid-derived suppressor cells (gMDSCs), **(f)** IL-10 expression upon mMDSCs, **(g)** IL-10 expression upon gMDSCs. The X-axis shows the timepoint at which the lungs were harvested for flow cytometry analysis. Data were plotted as Mean ± SEM and are shown as the frequency of $CD45^+$ population. gMFI stands for geometric mean fluorescence intensity and was used to define the expression of the individual markers upon the indicated cell types. gMFI was mostly used for low abundance cell surface markers and transcription factors. Statistical significance was calculated using a two-tailed student t-test considering unequal distribution. The exact p-values are provided in the Source Data file. *<0.05, **<0.01, ***<0.001, ****<0.0001. CFU stands for colony-forming units. NS stands for non-significant change, p-value was >0.05. The experiment was repeated two times.

Fig S15.

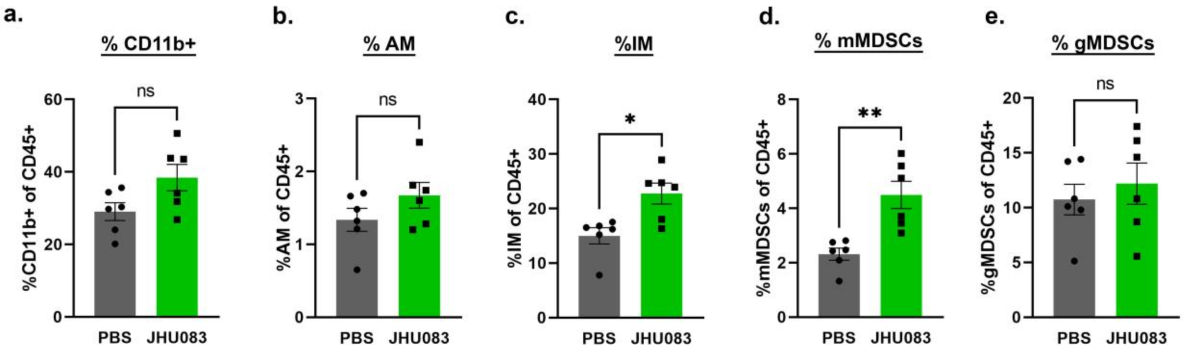

Fig S15. Effect of JHU083 administration upon myeloid cell subsets in uninfected 129S2 mice lungs at week 2. Uninfected 129S2 mice (n=6/group) were treated with JHU083 every day. The mice were sacrificed at week 2, and the lungs were harvested. Single cell suspensions of the lungs from all three groups were stained with appropriate antibodies and analyzed using multicolor-flow cytometry. We found no difference in the **(a)** CD11b+ myeloid cells and, **(b)** alveolar macrophages (AM). There was increased frequency of **(c)** interstitial macrophages (IM) and, **(d)** monocytic MDSC (mMDSCs) while **(e)** frequency of granulocytic MDSC (gMDSCs) remained unaltered. Data were plotted as Mean ± SEM and are shown as the frequency of $CD45^+$ population. Statistical significance was calculated using a two-tailed student t-test considering unequal distribution. The exact p-values are provided in the Source Data file. *<0.05. CFU stands for colony-forming units. NS stands for non-significant change, p-value was >0.05. The experiment was performed twice.

Fig S16.

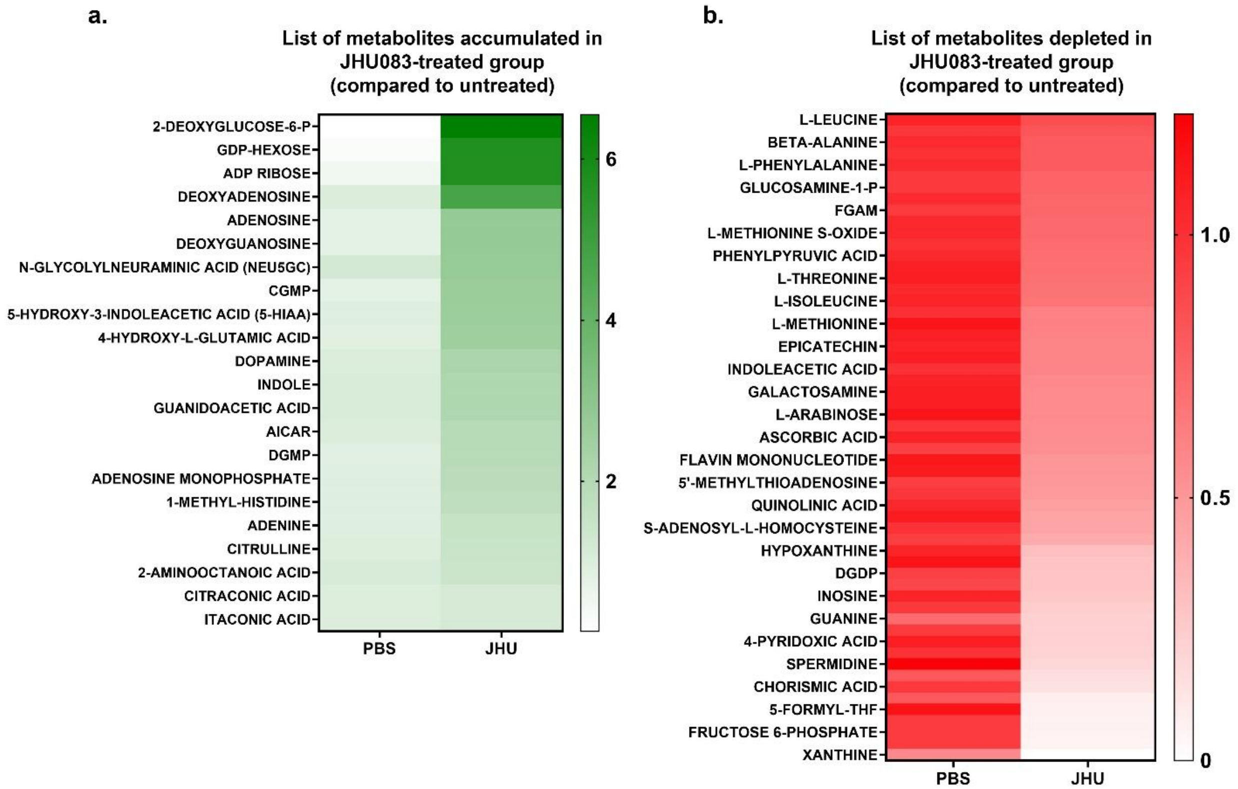

Fig S16. Heatmap representing the top metabolites that were either (a) enriched or (b) depleted in the lungs harvested from the animals treated with JHU083 (right-lane) compared to untreated group (left lane). The metabolites were methanol-extracted from the total lungs harvested at week 2 post infection and treatment (n=5/group). The metabolite abundance was normalized to the total lung tissue used for the extraction and untreated control. Statistical significance was calculated using a two-tailed student t-test considering unequal distribution. The exact p-values are provided in the Source Data file. The experiment was performed twice.

Fig S17.

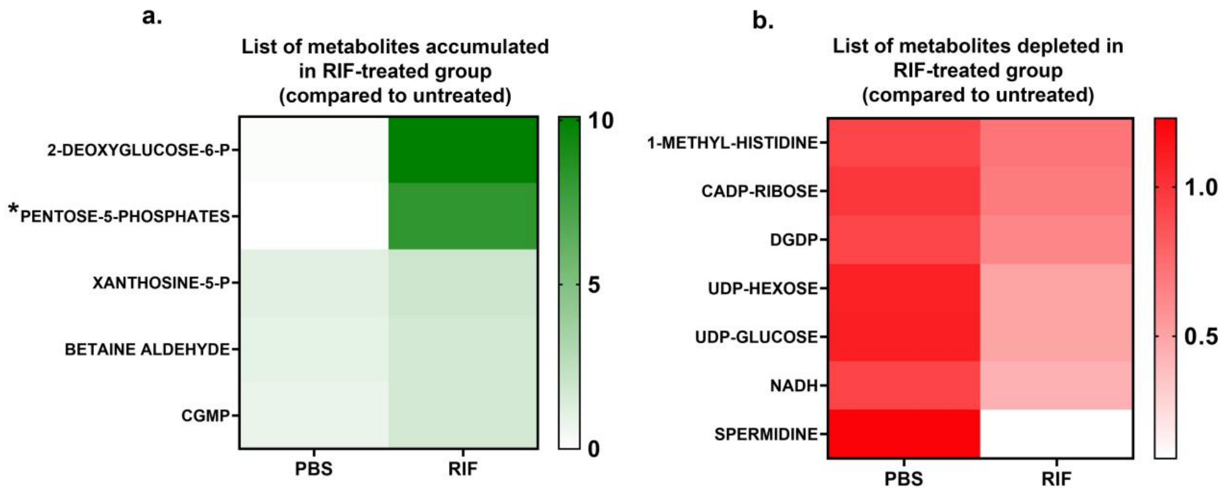

Fig S17. Heatmap representing the top metabolites that were either (a) enriched or (b) depleted in the lungs harvested from the animals treated with RIF (right-lane) compared to untreated group (left lane). The metabolites were methanol-extracted from the total lungs harvested at week 2 post infection and treatment (n=5/group). The metabolite abundance was normalized to the total lung tissue used for the extraction and untreated control. The exact p-values are provided in the Source Data file. Statistical significance was calculated using a two-tailed student t-test considering unequal distribution. *Pentose-5-phosphates include arabinose-5-phosphate, ribose-5-phosphate, and xylulose-5-phosphate. These three molecules have identical MW and RT and hence, are indistinguishable based on mass-spectrometry. The experiment was performed twice.

Fig S18.

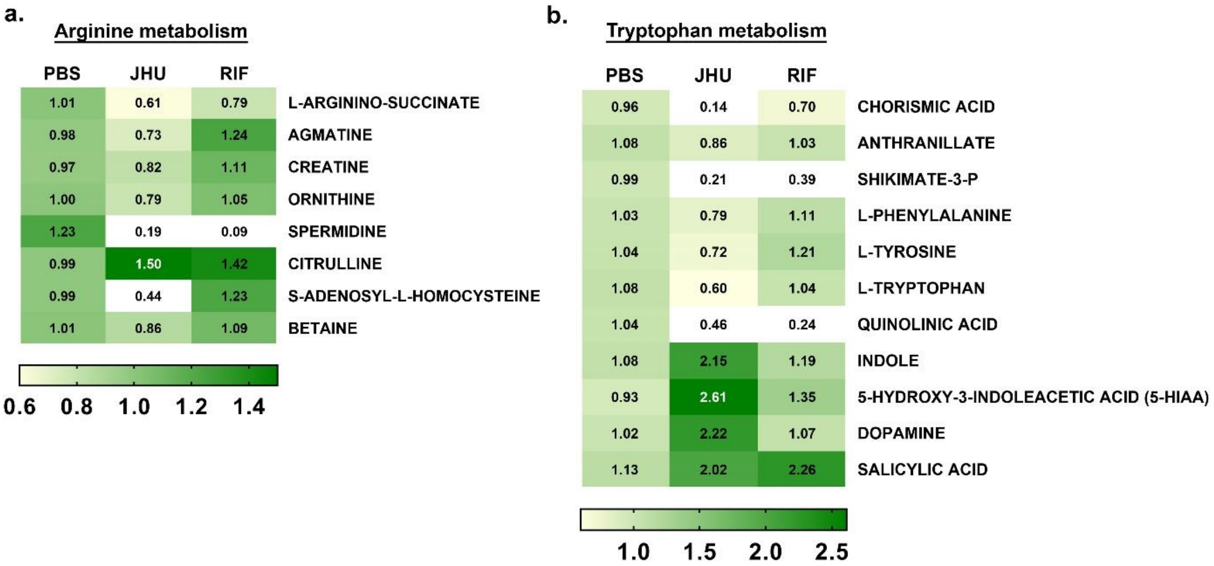

Fig S18. Heatmaps listing the metabolites that are affected by JHU083-treatment. As described in Fig 2a, *Mtb*-infected 129S2 mice were treated with JHU083 and RIF every day starting day 1 post-infection (n=5/group). Mice were sacrificed at week 2, the lungs were harvested, and total metabolites were methanol extracted as described in “Methods”. The total metabolites were normalized to the tissue weight and then to the untreated controls. We detected changes in the level of metabolites belonging to both **(a)** arginine and, **(b)** tryptophan metabolism pathways. Statistical significance was calculated using a two-tailed student t-test considering unequal distribution. The values in the cells correspond to the median value of the dataset. The experiment was performed once. The metabolomics data is provided as a separate excel sheet with normalized values along with the p-value calculations. The exact p-values are provided in the Source Data file. The experiment was performed twice.

Fig S19.

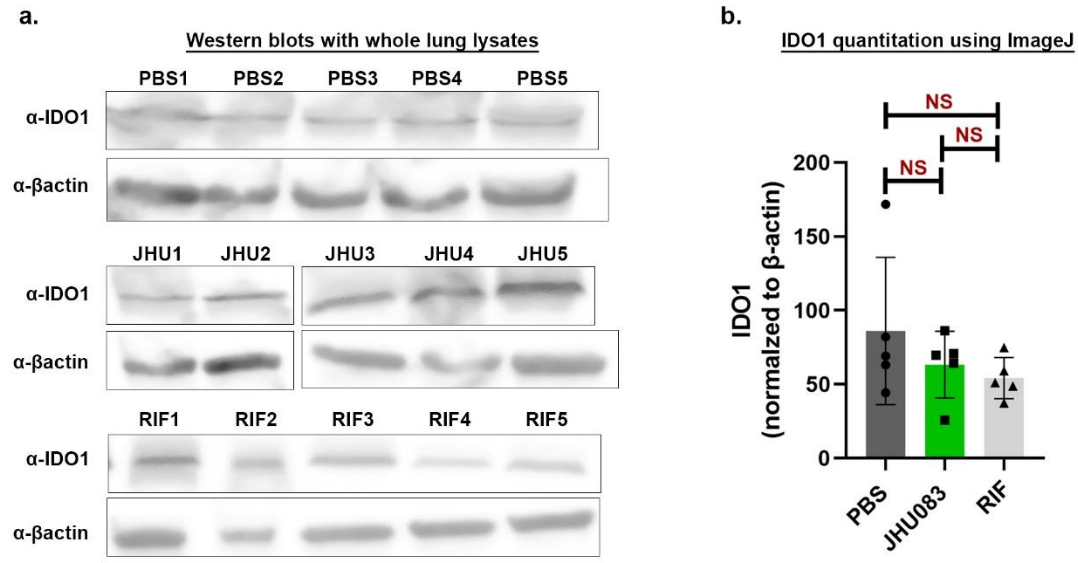

Fig S19. JHU083 administration does not alter the IDO1 levels in Mtb-infected lungs. As described in Fig 2a, six to 10 weeks old 129S2 female mice (n=5/group) were aerosol infected with ~200-300 CFU of *Mtb H37Rv*. The mice were treated with JHU083 or RIF via oral route one day after infection. 1 mg/Kg JHU083 was given daily for the first five days, and then the dose was reduced to 0.3 mg/Kg daily (M-F). The mice were sacrificed at week 2 post-infection/treatment. The lungs were harvested and homogenized to prepare whole lung lysate. Lung lysate corresponding to 10 µg protein was loaded per lane, electrophoresed, transferred to PVDF membrane. The level of IDO1 and β-actin was determined using specific primary antibody specified in the materials and methods section. **(a)** Western blot showing the IDO1 and β-actin levels in whole lung lysate from all three treatment groups. **(b)** Quantitation of IDO1 levels in the whole lung lysate using ImageJ based densitometry. Data were plotted as Mean ± SEM. Statistical significance was calculated using a two-tailed student t-test considering unequal distribution. The exact p-values are provided in the Source Data file. CFU stands for colony-forming units. NS stands for non-significant change, p-value was >0.05. The experiment was repeated twice.

Fig S20.

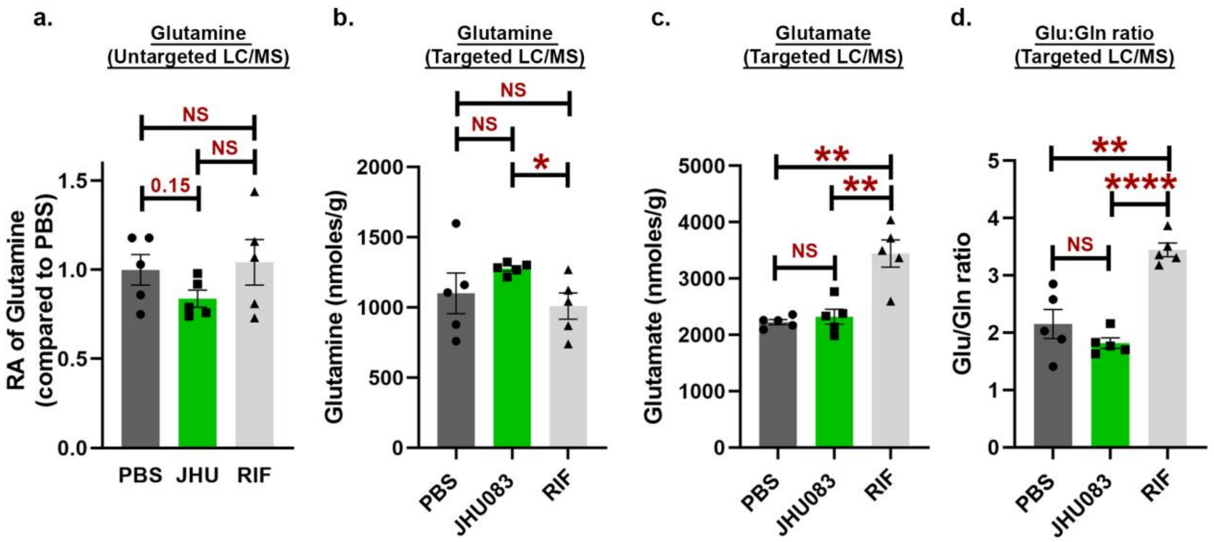

Fig S20. JHU083 treatment does not alter glutamine level in the *Mtb*-infected lungs. As described in Fig 2a, *Mtb*-infected 129S2 mice (n=5/group) were treated with JHU083 and RIF every day starting day 1 post-infection. Mice were sacrificed at week 2, the lungs were harvested, and total metabolites were methanol extracted as described in "Methods". The total metabolites were normalized to the tissue weight and then to the untreated controls. We detected no change in the level of **(a)** glutamine as per the untargeted metabolomics. We also observed no change in the levels of **(b)** glutamine, **(c)** glutamate and (**d)** glutamate and glutamine ratio, quantified as per targeted metabolomics. Data were plotted as Mean ± SEM. Statistical significance was calculated using a two-tailed student t-test considering unequal distribution. The exact p-values are provided in the Source Data file. *<0.05, **<0.01, ***<0.001, ****<0.0001. CFU stands for colony-forming units. NS stands for non-significant change, p-value was >0.05. The experiment was repeated twice.

LEGEND FOR TABLE

Supplementary Data 1: As described in Fig 2a, *Mtb*-infected 129S2 mice (n=5/group) were treated with JHU083 and RIF daily starting day 1 post-infection. Mice were sacrificed at weeks 2 and 5, the lungs were harvested, and total metabolites were extracted with methanol as described in "Methods." Sheet 1, labeled "Sample details," includes the description of all the samples that were used for the experiment. Sheet 2, labeled "Week 2_Normalized data" lists all the metabolites that were identified in the *Mtb*-infected lungs week 2 post-infection/treatment. The metabolite abundances were normalized to the tissue weight and then to the untreated controls. Data were plotted as Mean ± SEM. Statistical significance was calculated using a two-tailed student t-test considering unequal distribution. The exact p-values are provided in the table. Sheet 3, labeled "Week 5_Normalized data" lists all the metabolites that were identified in the *Mtb*-infected lungs, week 5 post-infection/treatment. The metabolite abundances were normalized to the tissue weight and then to the untreated controls. Data were plotted as Mean ± SEM. Statistical significance was calculated using a two-tailed student t-test considering unequal distribution. The exact p-values are provided in the table. Sheet 4, labeled "All metabolites" lists the metabolites that were expected to give a peak on the MS spectra. The values represent the area under the curve for individual metabolite peaks, present in *Mtb*-infected lungs weeks 2 and 5 post-infection/treatment. Zero indicates that we could not detect the specified metabolite peak in the corresponding sample. Sheet 5, labeled "Metabolites with signal peak" lists all the metabolites that were detected in the *Mtb*-infected lungs weeks 2 and 5 post-infection/treatment. The values represent the area under the curve for individual metabolite peaks, present in *Mtb*-infected lungs weeks 2 and 5 post-infection/treatment. on the corresponding mass spectra. Sheet 6, labeled "Metabolites with no signal peak" lists all the metabolites that could not be detected in the *Mtb*-infected lungs, weeks 2 and 5 post-infection/treatment. The values represent the area under the curve for individual metabolite peaks observed on the corresponding mass spectra.
