# Supplementary material for: Glutamine metabolism inhibition has dual immunomodulatory and antibacterial activities against Mycobacterium tuberculosis
Source: Nat Commun. 2023 Nov 16;14:7427. doi: 10.1038/s41467-023-43304-0 (PMC10654700; doi:10.1038/s41467-023-43304-0)
Supplement: Supplementary file 3 — Description of Additional Supplementary Files [file 41467_2023_43304_MOESM3_ESM.pdf]

Description of Additional Supplementary Files

Supplementary Data 1: As described in Fig 2a, *Mtb*-infected 129S2 mice (n=5/group) were 356 treated with JHU083 and RIF daily starting day 1 post-infection. Mice were sacrificed at weeks 357 2 and 5, the lungs were harvested, and total metabolites were extracted with methanol as 358 described in "Methods." (Sheet 1), labeled "Sample details," includes the description of all the 359 samples that were used for the experiment. (Sheet 2) labeled "Week 2_Normalized data" lists all 360 the metabolites that were identified in the *Mtb*-infected lungs week 2 post-infection/treatment. 361 The metabolite abundances were normalized to the tissue weight and then to the untreated 362 controls. Data were plotted as Mean ± SEM. Statistical significance was calculated using a two363 tailed student t-test considering unequal distribution. The exact p-values are provided in the 364 table. (Sheet 3) labeled "Week 5_Normalized data" lists all the metabolites that were identified in 365 the *Mtb*-infected lungs, week 5 post-infection/treatment. The metabolite abundances were 366 normalized to the tissue weight and then to the untreated controls. Data were plotted as Mean ± 367 SEM. Statistical significance was calculated using a two-tailed student t-test considering 368 unequal distribution. The exact p-values are provided in the table. (Sheet 4) labeled "All 369 metabolites" lists the metabolites that were expected to give a peak on the MS spectra. The 370 values represent the area under the curve for individual metabolite peaks, present in *Mtb*-infected 371 lungs weeks 2 and 5 post-infection/treatment. Zero indicates that we could not detect the 372 specified metabolite peak in the corresponding sample. (Sheet 5) labeled "Metabolites with signal 373 peak" lists all the metabolites that were detected in the *Mtb*-infected lungs weeks 2 and 5 post374 infection/treatment. The values represent the area under the curve for individual metabolite 375 peaks, present in *Mtb*-infected lungs weeks 2 and 5 post-infection/treatment. on the 376 corresponding mass spectra. (Sheet 6) labeled "Metabolites with no signal peak" lists all the 377 metabolites that could not be detected in the *Mtb*-infected lungs, weeks 2 and 5 post378 infection/treatment. The values represent the area under the curve for individual metabolite peaks 379 observed on the corresponding mass spectra.
