# Supplementary material for: Glutamine metabolism inhibition has dual immunomodulatory and antibacterial activities against Mycobacterium tuberculosis
Source: Nat Commun. 2023 Nov 16;14:7427. doi: 10.1038/s41467-023-43304-0 (PMC10654700; doi:10.1038/s41467-023-43304-0)
Supplement: Supplementary file 5 — Reporting Summary [file 41467_2023_43304_MOESM5_ESM.pdf]

nature portfolio

Corresponding author(s): William R Bishai

Last updated by author(s): Oct 24, 2023

Reporting Summary

Nature Portfolio wishes to improve the reproducibility of the work that we publish. This form provides structure for consistency and transparency in reporting. For further information on Nature Portfolio policies, see our Editorial Policies and the Editorial Policy Checklist.

Statistics

For all statistical analyses, confirm that the following items are present in the figure legend, table legend, main text, or Methods section.

n/a	Confirmed	
☐	☒	The exact sample size (*n*) for each experimental group/condition, given as a discrete number and unit of measurement
☐	☒	A statement on whether measurements were taken from distinct samples or whether the same sample was measured repeatedly
☐	☒	The statistical test(s) used AND whether they are one- or two-sided<br>*Only common tests should be described solely by name; describe more complex techniques in the Methods section.*
☐	☒	A description of all covariates tested
☐	☒	A description of any assumptions or corrections, such as tests of normality and adjustment for multiple comparisons
☐	☒	A full description of the statistical parameters including central tendency (e.g. means) or other basic estimates (e.g. regression coefficient) AND variation (e.g. standard deviation) or associated estimates of uncertainty (e.g. confidence intervals)
☐	☒	For null hypothesis testing, the test statistic (e.g. *F*, *t*, *r*) with confidence intervals, effect sizes, degrees of freedom and *P* value noted<br>*Give P values as exact values whenever suitable.*
☒	☐	For Bayesian analysis, information on the choice of priors and Markov chain Monte Carlo settings
☒	☐	For hierarchical and complex designs, identification of the appropriate level for tests and full reporting of outcomes
☒	☐	Estimates of effect sizes (e.g. Cohen's *d*, Pearson's *r*), indicating how they were calculated

Our web collection on statistics for biologists contains articles on many of the points above.

Software and code

Policy information about availability of computer code

Data collection	Metabolomics data was acquired with Compass HyStar 5.1 acquisition software and processed with TASQ 2022.
Data analysis	ImageScope open-source Software (Leica Biosystems) was used for granuloma quantification; Flow cytometry data were analyzed using FlowJo version 10.8.1 (Tree Star); Densitometric quantitation of the western blot bands was performed using ImageJ software (NIH); and statistical values were calculated using GradPad Prism software. Bishai Lab has purchased subscription to FlowJo and GraphPad Prism software. ImageScope and ImageJ are open-source software.

For manuscripts utilizing custom algorithms or software that are central to the research but not yet described in published literature, software must be made available to editors and reviewers. We strongly encourage code deposition in a community repository (e.g. GitHub). See the Nature Portfolio guidelines for submitting code & software for further information.

Data

Policy information about availability of data

All manuscripts must include a data availability statement. This statement should provide the following information, where applicable:

- Accession codes, unique identifiers, or web links for publicly available datasets
- A description of any restrictions on data availability
- For clinical datasets or third party data, please ensure that the statement adheres to our policy

The raw data generated during the study has been provided as a separate MS Excel spreadsheet labeled "Source Data file". Metabolomics dataset has been provided as "Supplementary Metabolomics Dataset". Any queries regarding the data should be addressed to the corresponding author.

Research involving human participants, their data, or biological material

Policy information about studies with human participants or human data. See also policy information about sex, gender (identity/presentation), and sexual orientation and race, ethnicity and racism.

Reporting on sex and gender	Not applicable
Reporting on race, ethnicity, or other socially relevant groupings	Not applicable
Population characteristics	Not applicable
Recruitment	Not applicable
Ethics oversight	Not applicable

Note that full information on the approval of the study protocol must also be provided in the manuscript.

Field-specific reporting

Please select the one below that is the best fit for your research. If you are not sure, read the appropriate sections before making your selection.

☒ Life sciences ☐ Behavioural & social sciences ☐ Ecological, evolutionary & environmental sciences

For a reference copy of the document with all sections, see nature.com/documents/nr-reporting-summary-flat.pdf

Life sciences study design

All studies must disclose on these points even when the disclosure is negative.

Sample size	No statistical method was used to predetermine sample size. Samples sizes were chosen based on the previous studies performed in the lab.
Data exclusions	No data points were excluded. For animal experiments, premature death of the mice or sample loss during tissue processing resulted in decreased sample size occasionally.
Replication	All experiments were performed at least two-times. 3-5 technical replicates for all cell culture-based experiments were used. All attempts at replication were successful.
Randomization	After aerosol infection, animals were randomly assigned into the individual treatment groups.
Blinding	The investigators were not blinded to the treatment groups given the long duration of the experiment, multiple individuals involved in drug dosing and distinguishable handling protocol for drugs used in the study.

Reporting for specific materials, systems and methods

We require information from authors about some types of materials, experimental systems and methods used in many studies. Here, indicate whether each material, system or method listed is relevant to your study. If you are not sure if a list item applies to your research, read the appropriate section before selecting a response.

Materials & experimental systems

n/a	Involved in the study
☐	☒ Antibodies
☒	☐ Eukaryotic cell lines
☒	☐ Palaeontology and archaeology
☐	☒ Animals and other organisms
☒	☐ Clinical data
☒	☐ Dual use research of concern
☒	☐ Plants

Methods

n/a	Involved in the study
☒	☐ ChIP-seq
☐	☒ Flow cytometry
☒	☐ MRI-based neuroimaging

Antibodies

Antibodies used

Document title: Antibodies used in the study

Antibody, Dilutions, Catalog, Supplier
APC/Fire™ 750 anti-mouse CD45 Ab, 1:1000, 103153, Biolegend
APC anti-mouse CD3 Ab, 1:250, 100235, Biolegend
BUV563 Rat Anti-Mouse CD4 Ab, 1:200, 612923, BD Biosciences
Alexa Fluor® 700 anti-mouse CD8a Ab, 1:200, 100730, Biolegend
OptiBuild™ BV650 Rat Anti-Mouse CD44, 1:200, 740455 ,BD Biosciences
Brilliant Violet 605™ anti-mouse CD62L Ab, 1:1000, 104437, Biolegend
BD Pharmingen™ Alexa Fluor® 647 Mouse anti-Bcl-6, 1:250, 561525, BD Biosciences
KLRG1 Monoclonal Ab (2F1), PE, eBioscience™ Ab, 1:500, 12-5893-82, ThermoFisher Scientific
PE anti-mouse IL-10 Ab, 1:200, 505007, Biolegend
Brilliant Violet 711™ anti-mouse TNF-α Ab, 1:200, 506349, Biolegend
Brilliant Violet 421™ anti-mouse IFN-γ Ab, 1:200, 505830, Biolegend
APC anti-mouse CD19 Ab 1:4000, 152409, Biolegend
PE/Cyanine7 anti-mouse/rat/human CD27 Ab, 1:1000, 124215, Biolegend
Brilliant Violet 605™ anti-mouse CD138 (Syndecan-1) Ab, 1:400, 142515, Biolegend
Brilliant Violet 650™ anti-mouse/human CD11b Ab, 1:250, 101239, Biolegend
Alexa Fluor® 647 anti-mouse F4/80 Ab, 123121, Biolegend
PE anti-mouse CD170 (Siglec-F) Ab, 1:1000, 155505, Biolegend
PerCP/Cyanine5.5 anti-mouse CD206 (MMR) Ab, 1:250, 141716, Biolegend
FITC anti-mouse CD86 Ab, 1:250, 105006, Biolegend
Brilliant Violet 605™ anti-mouse Ly-6G Ab, 1:500, 127639, Biolegend
PE/Cyanine7 anti-mouse Ly-6C Ab, 1:1600, 128017, Biolegend
BD Horizon™ BV711 Rat Anti-Mouse F4/80 Ab, 1:400, 565612, BD Biosciences
IDO (D8W5E) Rabbit mAb, 1:1000, 51851S, Cell Signaling Technology
HRP-conjugated β-actin Ab, 1:1000, 5125S, Cell Signaling Technology
Zombie Aqua™ Fixable Viability Kit*, 1:2000, 423101, Biolegend
*Not an antibody; Ab = Antibody

Validation

Only antibodies validated for specificity by the manufacturers were used.

Antibody Catalog Validation statement:
APC/Fire™ 750 anti-mouse CD45 Ab, 103153, As per manufacturer website, "Verified reactivity mouse; Clone 30-F11 reacts with all isoforms and both CD45.1 and CD45.2 alloantigens of CD45"

APC anti-mouse CD3 Ab, 100235, As per manufacturer website, "Verified reactivity mouse; Immunogen γδTCR-positive T-T hybridoma D1; Each lot of this antibody is quality control tested by immunofluorescent staining with flow cytometric analysis."

BUV563 Rat Anti-Mouse CD4 Ab, 612923, As per manufacturer website, "Reactivity; QC Testing Mouse." Reference for the publication using the Ab: Dialynas DP, Quan ZS, Wall KA, et al. Characterization of the murine T cell surface molecule, designated L3T4, identified by monoclonal antibody GK1.5: similarity of L3T4 to the human Leu-3/T4 molecule. J Immunol. 1983; 131(5):2445-2451. (Immunogen: Blocking, Depletion, Flow cytometry)

Alexa Fluor® 700 anti-mouse CD8a Ab, 100730, As per manufacturer website, "Verified reactivity mouse; Clone 53-6.7 antibody competes with clone 5H10-1 antibody for binding to thymocytes. The 53- 6.7 antibody has been reported to block antigen presentation via MHC class I and inhibit T cell responses to IL-2. This antibody has also been used for depletion of CD8a cells."

OptiBuild™ BV650 Rat Anti-Mouse CD44 740455, As per manufacturer website, "Verified reactivity mouse (Tested in Development); The IM7 antibody specifically recognizes an epitope on both alloantigens and all isoforms of the CD44 glycoprotein (Pgp-1, Ly-24).

Brilliant Violet 605™ anti-mouse CD62L Ab, 104437, As per manufacturer website, "Verified reactivity mouse"

BD Pharmingen™ Alexa Fluor® 647 Mouse anti-Bcl-6 561525, As per manufacturer website, "Verified Reactivity: Human (QC Testing), Mouse (Tested in Development)'

KLRG1 Monoclonal Ab, (2F1), PE, eBioscience™ Ab, 12-5893-82, As per manufacturer website, "Verified reactivity mouse"
PE anti-mouse IL-10 Ab, 505007, As per manufacturer website, "Verified reactivity mouse"

Brilliant Violet 711™ anti-mouse TNF-α Ab, 506349, As per manufacturer website, “Verified reactivity mouse”
Brilliant Violet 421™ anti-mouse IFN-γ Ab, 505830, As per manufacturer website, “Verified reactivity mouse”

APC anti-mouse CD19 Ab, 152409, As per manufacturer website, “Verified reactivity mouse”
PE/Cyanine7 anti-mouse/rat/human CD27 Ab, 124215, As per manufacturer website, “Verified reactivity mouse, rat, human”

Brilliant Violet 605™ anti-mouse CD138 (Syndecan-1) Ab, 142515, As per manufacturer website, “Verified reactivity mouse”
Brilliant Violet 650™ anti-mouse/human CD11b Ab, 101239, As per manufacturer website, “Verified reactivity Mouse, Human, Cynomolgus, Rhesus. Clone M1/70 has been verified for immunocytochemistry (ICC) and frozen immunohistochemistry (IHC-F)."

Alexa Fluor® 647 anti-mouse F4/80 Ab, 123121, As per manufacturer website, “Verified reactivity mouse”
PE anti-mouse CD170 (Siglec-F) Ab, 155505, As per manufacturer website, “Verified reactivity mouse”

PerCP/Cyanine5.5 anti-mouse CD206 (MMR) Ab, 141716, As per manufacturer website, “Verified reactivity mouse”. Clone C068C2 recognizes a region similar to clone MR5D3, based on the ability of the clones to block each other. Additional reported applications (for the relevant formats) include spatial biology (IBEX).
FITC anti-mouse CD86 Ab, 105006, As per manufacturer website, “Verified reactivity mouse”.

Brilliant Violet 605™ anti-mouse Ly-6G Ab, 127639, As per manufacturer website, “Verified reactivity mouse”.
PE/Cyanine7 anti-mouse Ly-6C Ab, 128017, As per manufacturer website, “Verified reactivity mouse”.

BD Horizon™ BV711 Rat Anti-Mouse F4/80 Ab, 565612, As per manufacturer website, “Verified Reactivity: Mouse (QC Testing)’
IDO (D8W5E) RAb,bit mAb, 51851S, As per manufacturer website, “IDO (D8W5E) Rabbit mAb recognizes endogenous levels of total IDO protein. This antibody does not cross-react with mouse IDO2. Species Reactivity: Mouse”

HRP-conjugated β-actin Ab, 5125S, As per manufacturer website, “Specificity / Sensitivity: β-Actin (13E5) Rabbit mAb (HRP Conjugate) detects endogenous levels of total β-actin protein. Depsite the high sequence identity between the cytoplasmic actin isoforms, β-actin and cytoplasmic γ-actin, β-Actin (13E5) Rabbit mAb (HRP Conjugate) #5125 does not cross-react with cytoplasmic γ-actin, or any other actin isoforms.; Species Reactivity: Human, Mouse, Rat, Monkey, Bovine, Pig; Species predicted to react based on 100% sequence homology: Hamster, Chicken, Dog, Horse, Rabbit

Animals and other research organisms

Policy information about studies involving animals; ARRIVE guidelines recommended for reporting animal research, and Sex and Gender in Research

Laboratory animals

129S2 mice: Six to ten weeks for infection studies; 3 to 4 months for BMDM assays (Charles River Laboratories, Wilmington, Massachusetts)
C3HeB/FeJ mice = Six to ten weeks (Jackson Laboratories, Bar Harbor, Massachusetts)
C3H mice = Six to ten weeks (Jackson Laboratories, Bar Harbor, Massachusetts)
Balbc SCID mice = Six to ten weeks (Jackson Laboratories, Bar Harbor, Massachusetts)
C57BL/6 = 3 to 4 months (Jackson Laboratories, Bar Harbor, Massachusetts)

All animals were housed in individually-ventilated cages under 12 h dark/light cycle in room with ambient temperature between 20-24? and a relative humidity of 45 to 65%. Each cage contained no more than five mice of the same strain.

Wild animals

The study did not involve wild animals

Reporting on sex

Only female mice were used for the studies for easier co-housing and to keep the managable number of animals per group.

Field-collected samples

The study did not involve field collected samples

Ethics oversight

All animal studies were performed per the protocols approved by the Johns Hopkins Animal Care and Use Committee of the Johns Hopkins School of Medicine. All animal were procured and handled as per the approved mouse protocol number M022M36.

Note that full information on the approval of the study protocol must also be provided in the manuscript.

Plants

Seed stocks

Report on the source of all seed stocks or other plant material used. If applicable, state the seed stock centre and catalogue number. If plant specimens were collected from the field, describe the collection location, date and sampling procedures.

Novel plant genotypes

Describe the methods by which all novel plant genotypes were produced. This includes those generated by transgenic approaches, gene editing, chemical/radiation-based mutagenesis and hybridization. For transgenic lines, describe the transformation method, the number of independent lines analyzed and the generation upon which experiments were performed. For gene-edited lines, describe the editor used, the endogenous sequence targeted for editing, the targeting guide RNA sequence (if applicable) and how the editor was applied.

Authentication

Describe any authentication procedures for each seed stock used or novel genotype generated. Describe any experiments used to assess the effect of a mutation and, where applicable, how potential secondary effects (e.g. second site T-DNA insertions, mosiacism, off-target gene editing) were examined.

Flow Cytometry

Plots

Confirm that:

☒ The axis labels state the marker and fluorochrome used (e.g. CD4-FITC).

☒ The axis scales are clearly visible. Include numbers along axes only for bottom left plot of group (a 'group' is an analysis of identical markers).

☒ All plots are contour plots with outliers or pseudocolor plots.

☒ A numerical value for number of cells or percentage (with statistics) is provided.

Methodology

Sample preparation	Single-cell suspension preparation: For multicolor flow cytometry analysis, lungs and spleens were collected in 5 ml MACS Tissue storage solution (Cat: 130-100-008; Miltenyi Biotec, Gaithersburg, MD) followed by storage at 4 °C until processing. The lungs were dissected into individual lobes, and single-cell suspension was prepared using the mouse lung dissociation kit (Cat: 130-095-927; Miltenyi Biotec, Gaithersburg, MD) and GentleMACS™ Dissociator treatment following the manufacturer's protocol. Spleens were mechanically dissociated in digestion buffer (RPMI 1640 + 10% FBS + 0.2 mg/ml collagenase D + DNAse). Both lung and spleen cells were then incubated with ACK lysis buffer at RT for 2-5 minutes to lyse the red blood cells. The cell suspension was then washed with RPMI complete media and resuspended in the appropriate volume of the same media. Trypan blue staining was performed to assess the viability of the cell suspensions.<br><br>Multicolor flow cytometry: Lung single-cell suspensions were incubated with TruStain FcX™ (anti-mouse CD16/32) antibody (Cat: 101320; BioLegend, San Diego, CA) in eBioscience™ Flow Cytometry Staining Buffer (Cat: 00-4222-57, San Diego, CA) for 20 min at room temperature to block non-specific antibody binding. The cells were then incubated with appropriate antibody cocktails and fixation buffer (Cat: 420801; BioLegend). For intracellular staining, the cells were stained using True-Nuclear™ Transcription Factor Buffer Set (Cat: 424401; Biolegend) following the manufacturer's protocol. Intracellular cytokine stimulation and staining was performed as described earlier 72. Briefly, lung cells were incubated with cell activation cocktail (Biolegend) and monensin for 4 h. The cytokine staining was performed using Cyto-Fast™ Fix/Perm Buffer Set (Cat: 426803; BioLegend) following the manufacturer's protocol. The stained cells were stored in Cyto-Last™ Buffer (Cat: 422501; BioLegend) at 4 °C till the data acquisition.
Instrument	The data were acquired on BD LSRFortessa™ Cell Analyzer (BD Biosciences, San Jose, CA).
Software	the data were analyzed using FlowJo version 10.8.1 (Tree Star)
Cell population abundance	FSC and SSC gating was performed to remove the aggregates, dead cells and doublets. The singlets were further gated for live cells. Most cell populations were expressed as the frequency of total immune cells (CD45+). For low abundance markers, geometrical mean of fluorescence (gMFI) was used.
Gating strategy	Gating strategies are provided in the supplementary figures S6, S7, S8, S12 and S13. Instead of beads, splenocytes were used for single cell stain controls and creating compensation matrices.

☒ Tick this box to confirm that a figure exemplifying the gating strategy is provided in the Supplementary Information.
